# Development of a Sweetness Sensor for Aspartame, a Positively Charged High-Potency Sweetener

**DOI:** 10.3390/s140407359

**Published:** 2014-04-23

**Authors:** Masato Yasuura, Yusuke Tahara, Hidekazu Ikezaki, Kiyoshi Toko

**Affiliations:** 1 Graduate School of Information Science and Electrical Engineering, Kyushu University, 744 Motooka, Nishi-ku, Fukuoka 819-0395, Japan; 2 Faculty of Information Science and Electrical Engineering, Kyushu University, 744 Motooka, Nishi-ku, Fukuoka 819-0395, Japan; E-Mails: tahara@belab.ed.kyushu-u.ac.jp (Y.T.); toko@ed.kyushu-u.ac.jp (K.T.); 3 Intelligent Sensor Technology, Inc., 5-1-1 Onna, Atsugi-shi, Kanagawa 243-0032, Japan; E-Mail: Ikezaki.Hidekazu@insent.co.jp; 4 Research and Development Center for Taste and Odor Sensing, Kyushu University, 744 Motooka, Nishi-ku, Fukuoka 819-0395, Japan

**Keywords:** taste sensor, high-potency sweetener, sweetness sensor, aspartame, lipid/polymer membrane

## Abstract

Taste evaluation technology has been developed by several methods, such as sensory tests, electronic tongues and a taste sensor based on lipid/polymer membranes. In particular, the taste sensor can individually quantify five basic tastes without multivariate analysis. However, it has proven difficult to develop a sweetness sensor, because sweeteners are classified into three types according to the electric charges in an aqueous solution; that is, no charge, negative charge and positive charge. Using membrane potential measurements, the taste-sensing system needs three types of sensor membrane for each electric charge type of sweetener. Since the commercially available sweetness sensor was only intended for uncharged sweeteners, a sweetness sensor for positively charged high-potency sweeteners such as aspartame was developed in this study. Using a lipid and plasticizers, we fabricated various lipid/polymer membranes for the sweetness sensor to identify the suitable components of the sensor membranes. As a result, one of the developed sensors showed responses of more than 20 mV to 10 mM aspartame and less than 5 mV to any other taste. The responses of the sensor depended on the concentration of aspartame. These results suggested that the developed sweetness sensor had high sensitivity to and high selectivity for aspartame.

## 1. Introduction

The taste-sensing of humans chiefly consists of the five basic tastes, that is, saltiness, sourness, sweetness, bitterness and umami [1,2]. Each basic taste has own role as a biological signal. Saltiness indicates the existence of minerals, which are good indicators of the electrolyte balance in foods and beverages. Sourness and bitterness indicate the risk of rottenness and poison, respectively. Sweetness indicates nutrient sources such as sugars. Umami is a signal of proteins and amino acids. These basic tastes are perceived on sensory organs, called taste buds, on the tongue. Each taste bud has about 50–150 taste-receptor cells. The mechanisms of taste-sensing on taste-receptor cells have been investigated by various approaches [1–4], for example, by biological methods, behavioral assays and molecular theories. In particular, the discovery of sweetness receptors and umami receptors contributed a lot to clarify the mechanisms of sweetness and umami taste perception. These receptors have broad selectivity to the corresponding tastes and can detect substances with diverse chemical structures despite having only one type of heterodimeric receptor in each cell [3–7].

Sweet substances include a large number of compounds with various chemical structures and sizes, for example, sugars (glucose), alditols (mannitol), peptides (aspartame), D-amino acids (D-alanine), sulfonyl amides (acesulfame potassium) and proteins (monellin). Two types of G-protein-coupled receptor (T1R2 and T1R3) compose a heterodimeric receptor, which acts as a sweetness receptor. T1R2+T1R3 heterodimeric receptors respond to sweeteners with all chemical structures. Although the AH-B theory is one of the most widely accepted models of the sweeteners' structural features, no one can explain the common structural features among only sweeteners [8–11]. The number of times that a sweetener is sweeter than sucrose is called sweetener potency. The potency of a sweetener is compared with sucrose mainly in the threshold levels of the sweetener and sucrose. Sugars and alditols, such as glucose and mannitol, are considered low-potency sweeteners, whose sweetener potencies are about 1 and less.

**Figure 1.**
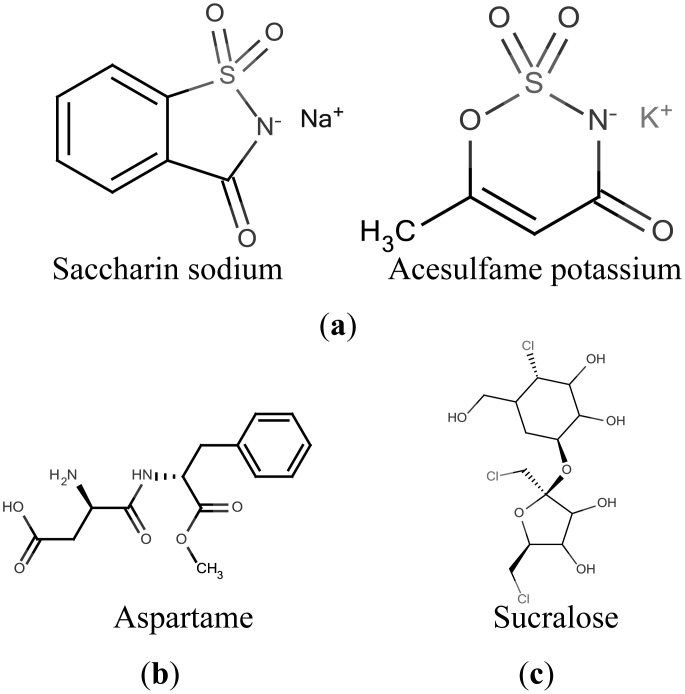
Four typical high-potency sweeteners. They are classified in three types by the electric charge under acidic conditions. (**a**) Negatively charged high-potency sweeteners; (**b**) Positively charged high-potency sweeteners; (**c**) No electrical charge high-potency sweeteners.

On the other hand, sweeteners with a sweetener potency exceeding 10 are defined as high-potency sweeteners, for example, acesulfame potassium and aspartame (Figure 1). Intriguingly, at very high concentrations, low-potency sweeteners, such as sucrose, display higher sweetness intensity than high-potency sweeteners. Hence, low-potency sweeteners are also called high-intensity sweeteners [9,11–13]. T1R2 or T1R3 knockout (KO) animals mainly exhibit no response to sweeteners physiologically or behaviorally. A surprising exception is that T1R2+T1R3 and T1R3 only receptors exhibit responses to very high concentrations (over 300 mM) of natural sugars despite the other receptor KO. In addition, both T1R2 and T1R3 KO animals physiologically and behaviorally exhibit no response to high concentrations of natural sugars [1,3].

Sensory evaluation, which is a type of test using human sensory systems, has been carried out to estimate the tastes of samples [14,15]. In sensory evaluations of foods, beverages or pharmaceutical products, well-trained sensory panelists evaluate samples by actually tasting them. Hence, sensory evaluations have several problems, for example, low objectivity, low reproducibility, the stress possibly imposed on panelists and the significant cost of selecting and training panelists. Additionally, in the medical and pharmaceutical industries, it is difficult to carry out sensory evaluations because of the potential for medication side effects. Although quantitative analysis can be conducted, it cannot be used to estimate the intensities of each basic taste. Against these backgrounds, objective methods of evaluating tastes without using human sensory systems have attracted attention. The development of objective methods of evaluating taste should contribute greatly to the compliance of drug products and the qualities of foods and beverages.

Examinations of objective methods of taste evaluation, such as electronic tongues (e-tongues), have been performed worldwide [16–23]. Since e-tongues are potentiometric multisensor systems mostly using metal and ion-selective electrodes, principal component analysis (PCA) and partial least squares (PLS) analysis are generally carried out to analyze taste information obtained by sensor outputs with low selectivity. Objective evaluations of the taste of unknown samples are difficult, because PCA needs to define the meanings of each principal component. In spite of this issue, e-tongues are suitable for comparing and distinguishing known samples, such as for quality control. There have been reports on the assessment of the bitterness of drugs and bitterness-masking formulations using e-tongues [16,18,20,23]. Rundnitskaya *et al.* [23] mentioned the quantification of bitterness of structurally various active pharmaceutical ingredients using an e-tongue under parameter-limited conditions.

Our research team has developed a taste sensor, which is an e-tongue with global selectivity, using some electrodes with lipid/polymer membranes comprising a lipid, polyvinyl chloride (PVC), and a plasticizer as sensing parts [24–29]. Global selectivity is one of the unique characteristics of our taste sensor. This means that the taste sensor must respond consistently to the same taste similarly to the human tongue, despite the various chemical structures and sizes of tastants. The taste sensor has been commercialized by Intelligent Sensor Technology, Inc., (Kanagawa, Japan) as a taste sensing system and is the first e-tongue system commercialized in the world. Each taste sensor electrode in the sensor system has global selectivity, responding to only one taste. The taste-sensing system is a potentiometric measurement system, which determines the membrane potential of lipid/polymer membranes. The change in membrane potential is used as the sensor output. It is caused by electrical and hydrophobic interactions between the lipid/polymer membrane and tastants in a sample solution. The taste-sensing system can quantify each basic taste intensity of foods and beverages from the change in each membrane potential. The global selectivity of this sensing method is based on the common characteristics of each basic taste substance, for example, bitterness: high hydrophobicity, sourness: proton donors, saltiness: metal cations. The taste sensor system is used in the food, beverage and pharmaceutical industries. Some products developed by these industries using the taste sensor system are now in common use [30].

The taste sensor system can quantify the intensities of each basic taste by the membrane potential measurement. Because of the measurement principle, it is difficult to evaluate sweetness using only one sensor electrode. Since sweet substances consist of nonelectrolytes (sugars), positively charged electrolytes (peptides) and negatively charged electrolytes (sulfonyl amides) under acidic conditions (most food environments), three types of sweetness sensor membrane are required for each electric charge type of sweetener. The sensor in the taste sensing system for nonelectrolytes (sugars and sugar alcohols) has already been developed and commercialized as a sweetness sensor [31,32]. The commercially available sweetness sensor is used in the food, beverage and pharmaceutical industries to estimate the sweet taste intensity of sugars and sugar alcohols. As mentioned above, in principle, it is difficult to develop a sweetness sensor for all sweet substances. Hence, we decided to develop two additional types of sweetness sensor, that is, for positively charged sweeteners (peptides) and for negatively charged sweeteners (sulfonyl amides). Both positively and negatively charged electrolyte sweeteners are mainly included in high-potency sweeteners. Such sweeteners have recently been used as sweeteners in low-calorie diets and bitterness-masking ingredients in pharmaceutical products, and are commonly used in the food, beverage and pharmaceutical industries [12–15,29,33–35]. In this study, a sweetness sensor for aspartame, one of positively charged high-potency sweeteners was developed with high selectivity and the capability of quantifying sweetness. Aspartame is one of the top six high-potency sweeteners, which hold almost the entire share of the global market for high-potency sweeteners.

## 2. Experimental Section

### 2.1. Lipid/Polymer Membrane

A lipid/polymer membrane, comprising a lipid, PVC and a plasticizer, works as both a recognition element and a transducer in the taste-sensing system. The responses of a lipid/polymer membrane to each basic taste depend on the concentrations and combination of the lipid and plasticizer. A taste sensor with global selectivity is realized using this characteristic.

A lipid/polymer membrane is positively or negatively charged on its surface in an aqueous solution. As is the case for a sample solution including electrolyte tastants (saltiness, sourness and umami substances), electrically charged tastants electrically interact with and are adsorbed on an oppositely charged lipid/polymer membrane, and cause the change in membrane potential. As is the case for a sample solution containing hydrophobic tastants (bitterness and astringent substances), the tastants electrically and hydrophobically interact with a sensor membrane and are adsorbed onto the surface of the membrane. Hence, the interaction between tastants and the membrane is stronger than that without hydrophobic interaction, often inducing a change in membrane potential exceeding that expected from the Nernst equation.

**Figure 2.**
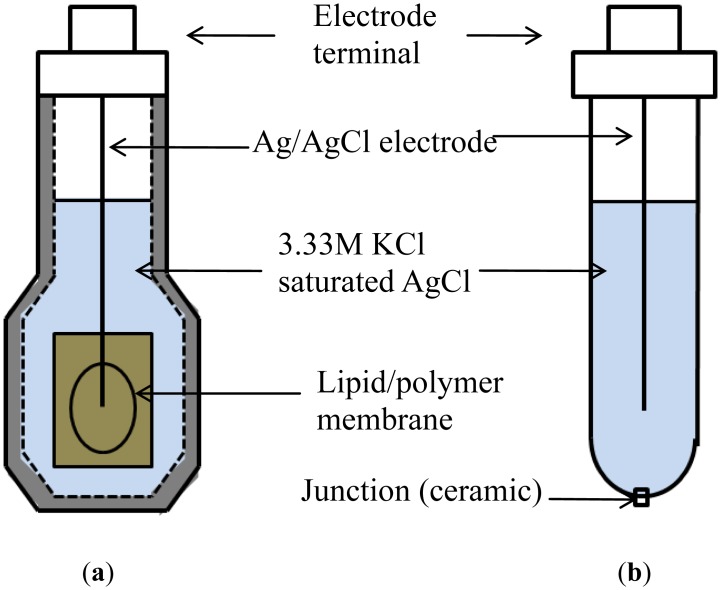
Two kinds of electrode are used in the taste-sensing system. (**a**) Sensor electrode; (**b**) Reference electrode.

The membrane potential is determined as the voltage between the sensor electrode and a reference electrode (Figure 2). The change in the membrane potential is calculated as the differential value between the membrane potentials in a reference solution and a sample solution. In this study, to determine suitable conditions for a lipid/polymer membrane to exhibit high sensitivity and selectivity to positively charged sweeteners, the quantities and types of the membrane components (lipid and plasticizer) were varied to adjust the hydrophobicity and the electric charge of the membrane surface.

### 2.2. Measurement Procedure

**Figure 3.**
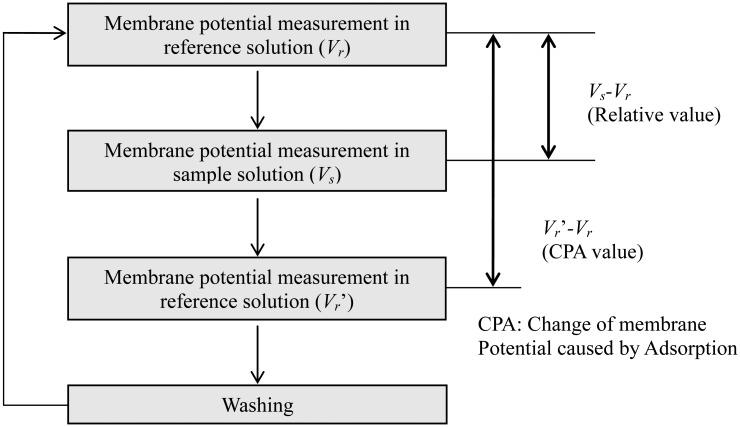
Measurement procedure of taste sensing.

Lipid/polymer membranes were made by means of a conventional protocol [24,25,27]. Usually, the fabricated membranes using this protocol can be used about 3000 times [27]. Measurements were carried out using the TS-5000Z taste-sensing system (Intelligent Sensor Technology, Inc., (Kanagawa, Japan). The measurement procedure was as follows (Figure 3) [24,25,27]. First, the membrane potential for a reference solution (30 mM KCl, 0.3 mM tartaric acid), Vr, was measured by potentiometry between sensor electrodes and a reference electrode (Ag/AgCl electrode). Second, the membrane potential for a sample solution, Vs, was determined. The difference between Vs and Vr, that is, Vs-Vr, is defined as a relative value. Then, the membrane potential for the reference solution was measured again (Vr'), and the difference between Vr' and Vr, that is, Vr'-Vr, was called the CPA value (change in membrane potential caused by adsorption) [24,25,27]. Finally, the membrane was rinsed with a sensor-rinsing solution (30 vol% ethanol, 100 mM KCl and 10 mM KOH). This procedure was repeated five times for each sample, and the average of the relative or CPA values was used as the relative or CPA value of each sample, respectively.

### 2.3. Selection of Plasticizers

**Figure 4.**
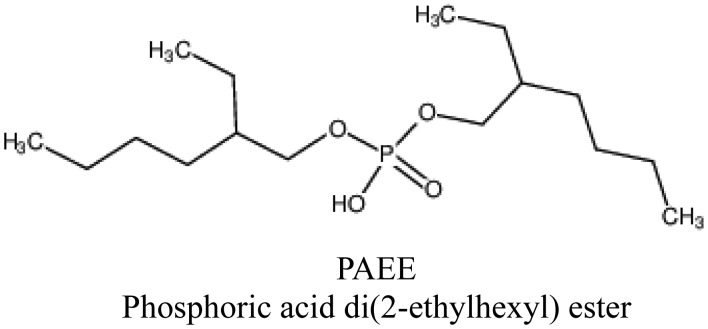
Phosphoric acid di(2-ethylhexyl) ester (PAEE). PAEE is often used in lipid/polymer membranes for taste sensor as a lipid.

**Figure 5.**
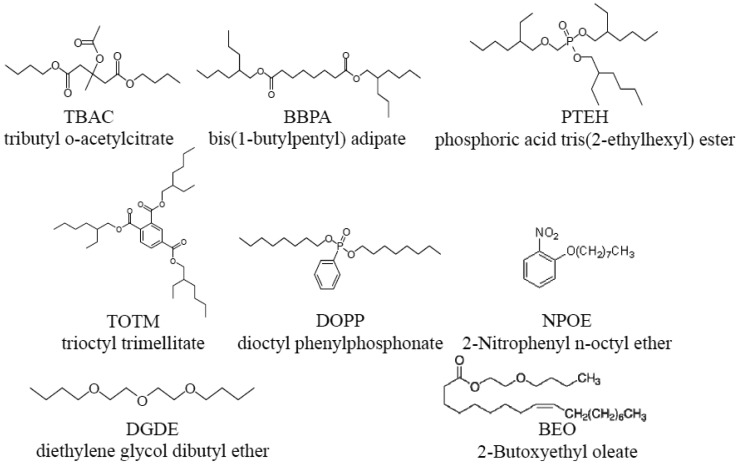
Plasticizers: eight plasticizers were used in this study.

Lipid/polymer membranes comprising phosphoric acid di(2-ethylhexyl) ester (PAEE, Tokyo Chemical Industry Co., Ltd., Tokyo, Japan, Figure 4) as a lipid, PVC and a plasticizer were fabricated. PAEE is one of the lipids used in the commercially available taste sensors. This lipid has negative charges in an aquous solution. The plasticizers used (Figure 5) were as follows: tributyl O-acetyl citrate (TBAC, Tokyo Chemical Industry Co., Ltd., Tokyo, Japan), bis(1-butylpentyl) adipate (BBPA, Sigma-Aldrich Co. LLC., St. Louis, MO, USA), phosphoric acid tris(2-ethylhexyl) ester (PTEH, Tokyo Chemical Industry Co., Ltd., Tokyo, Japan), trioctyl trimelitate (TOTM, Tokyo Chemical Industry Co., Ltd., Tokyo, Japan), dioctyl phenyl phosphonate (DOPP, Dojindo Molecular Technologies, Inc., Kumamoto, Japan), 2-nitrophenyl n-octyl ether (NPOE, Dojindo Molecular Technologies, Inc.), diethylene glycol dibutyl ether (DGDE, Sigma-Aldrich Co. LLC., St. Louis, MO, USA) and 2-butoxyethyl oleate (BEO, Dojindo Molecular Technologies, Inc., Kumamoto, Japan). The quantities of the lipid and a type of plasticizer contained were the same in each membrane. These eight types of lipid/polymer membrane (an amount of PAEE × 8 types of plasticizer) were used as the sensor membranes of eight sensor electrodes to measure basic taste samples (Table 1). Since the CPA values generally had higher selectivity than the relative values because of the unresponsiveness to electrolyte tastants (saltiness, sourness and umami substances) [24–27,36,37], we focused our attention on the CPA values rather than the relative values in this study. In addition, three required specifications are aimed at in terms of the commercial availability of the sensor as a sweetness sensor for positively charged sweeteners. One of the requirements was a response of at least 20 mV to 10 mM aspartame in terms of the CPA value. Another requirement was not to respond to any other basic tastes (high selectivity). The other was the concentration dependence of the responses to aspartame.

### 2.4. Effect of Lipid Quantity on CPA Values (the First Stage)

Eight types of sensor membrane with PAEE concentrations of 0–500 mg were fabricated to investigate the effect of the PAEE concentration on sensor responses. The membranes comprising PAEE, PVC and a plasticizer, chosen on the basis of the results of Section 2.3, were used as the sensor membranes. The basic taste samples were measured using eight sensor electrodes in each sensor membrane.

### 2.5. Effect of Lipid Quantity on CPA Values (the Second Stage)

Three types of lipid/polymer membrane with PAEE concentrations of 500–1000 mg were fabricated to investigate the effect of the high PAEE concentration on sensor responses and the aspartame concentration dependence of sensor responses. The membranes were composed of PAEE, PVC and the plasticizer, chosen on the basis of the results of Section 2.3. They were used as the sensor membranes of eight sensor electrodes to measure basic taste samples and four concentrations of aspartame samples. There were 5 concentrations (0.1–10 mM) of aspartame in total samples. Each aspartame sample included 30 mM KCl and 0.3 mM tartaric acid (the reference solution components).

## 3. Results and Discussion

### 3.1. Selection of Plasticizers

**Figure 6.**
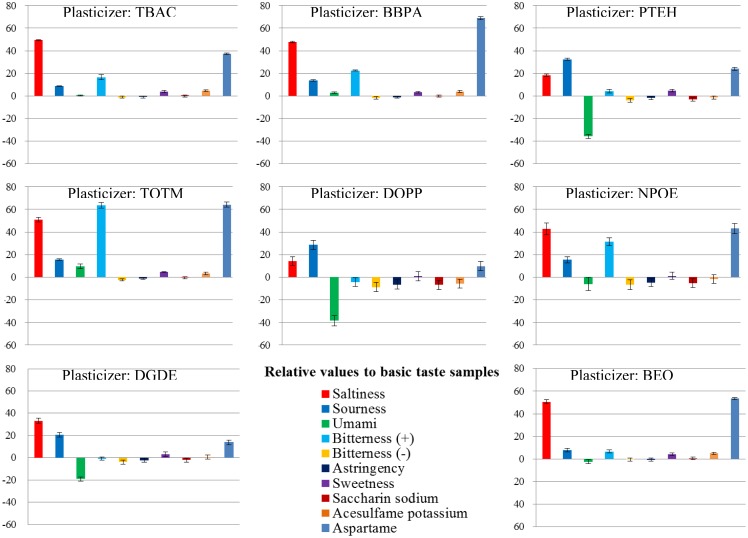
Relative values: basic taste samples were measured using eight kinds of lipid/polymer membrane (parameter of membranes: type of plasticizer).

**Figure 7.**
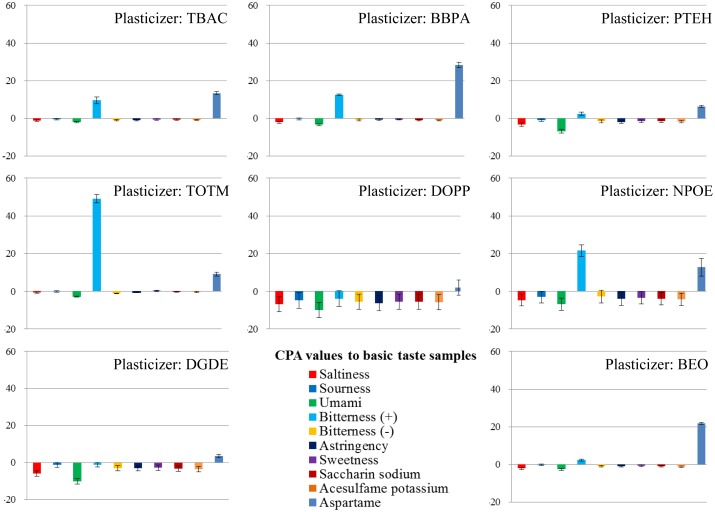
CPA values: basic taste samples were measured using 8 kinds of lipid/polymer membrane (parameter of membranes: type of plasticizer).

To investigate the patterns and ratios of membrane components suitable for the sweetness sensor, we used PAEE as a lipid, PVC as a polymer and eight types of plasticizer. First, we examined which plasticizer was the most suitable. Using each plasticizer with PAEE in each membrane, we fabricated eight lipid/polymer membranes and measured the basic taste samples using them. The results of the measurements are shown in Figures 6 and 7. Most of the basic tastes mostly showed negligible CPA values. Three of them (saltiness, sourness and umami) often showed large relative values. This appears to be caused by the low hydrophobicity of these tastants. In general, high CPA values are caused by tastants with high hydrophobicity. Bitterness (–), astringency, saccharin sodium and acesulfame potassium (Table 1) mostly showed negligible relative and CPA values. These tastants had negative electric charges in sample solutions and evoked repulsive electrostatic interactions with negatively charged sensor membranes. Sucrose (sweetness sample) also exhibited negligible relative and CPA values because of the uncharged substance. Without the adsorption of tastants, these five samples exhibited negligible responses. Hence, only two samples, bitterness (+) and aspartame, often exhibited high CPA values.

**Figure 8.**
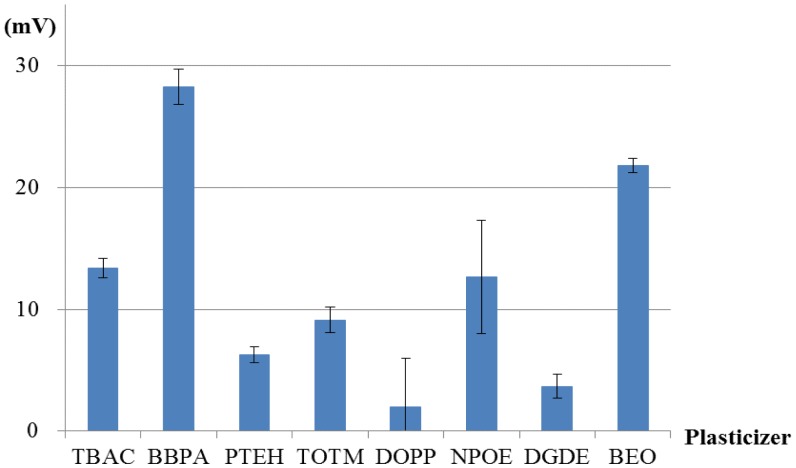
The responsivity in terms of CPA values of the membranes comprising PAEE, PVC and one of the eight types of plasticizer to 10 mM aspartame. Aspartame sample was measured using 8 kinds of lipid/polymer membrane (parameter: type of plasticizer)

**Figure 9.**
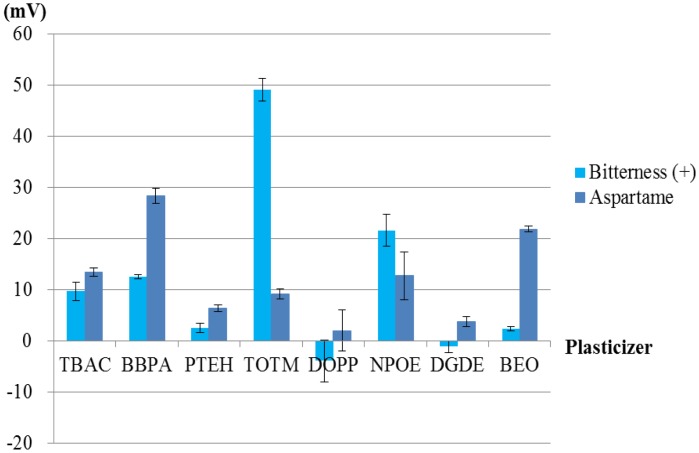
The selectivity of the membranes comprising PAEE, PVC and one of the eight types of plasticizer in terms of CPA values. Bitterness (+) and aspartame samples were measured using eight kinds of lipid/polymer membrane (parameter: type of plasticizer).

**Figure 10.**
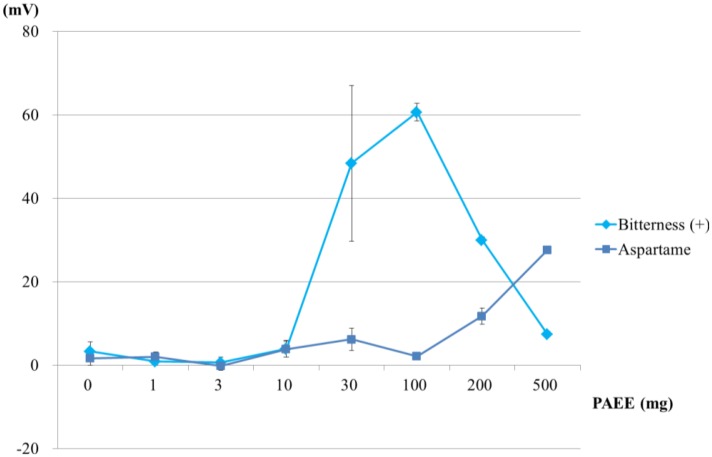
CPA values of membranes containing PAEE (0–500 mg) and BEO. Bitterness (+) and aspartame samples were measured using eight kinds of lipid/polymer membrane (parameter: lipid quantity).

**Figure 11.**
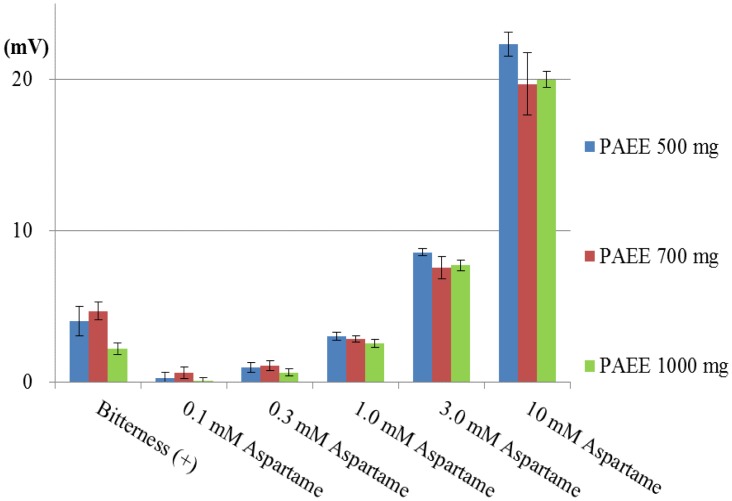
CPA values of membranes containing PAEE (500–1000 mg) and BEO. Bitterness (+) and five concentrations of aspartame samples were measured using three kinds of lipid/polymer membrane (parameter of membranes: lipid quantity).

Figures 8, 9, 10 and 11 show only bitterness (+) and aspartame because of the negligible CPA values of the others. The responsivity to the aspartame sample in terms of the CPA values was as follows (Figure 8). Two membranes including BBPA or BEO as a plasticizer met the requirement of a response of at least 20 mV to 10 mM aspartame. The selectivity of each membrane is shown in Figure 9. The membrane using the plasticizer BBPA did not meet the selectivity requirement because of the high CPA value to bitterness (+). On the other hand, the membrane including the plasticizer BEO indicated high selectivity. Since there was no interfering taste, the plasticizer BEO was chosen as the plasticizer in the sensor for positively charged sweeteners in this study.

### 3.2. Effect of Lipid Quantity on CPA Values (the First Stage)

Next, we investigated the effect of the quantity of PAEE on CPA values. Eight sensor membranes comprising 0–500 mg PAEE, PVC and BEO were fabricated. The basic taste samples were measured using these membranes. The responses of these membranes to bitterness (+) and aspartame are shown in Figure 10. The response to bitterness (+) was greatest for the PAEE quantity of 100 mg. The responses to bitterness (+) increased monotonically with increasing the quantity of PAEE (10–100 mg) and decreased monotonically with increasing the quantity of PAEE (100–500 mg). Generally, a peak in the lipid quantity dependence of relative or CPA values is caused by two principles, as discussed in previous papers [26,37]. When the lipid quantity increases, the increasing electric charges of the membranes causes an increase in the adsorption of tastants by their electrical interaction. Simultaneously, decreasing the hydrophobicity of the membrane causes the change in intensity of the hydrophobic interaction between the sensor membranes and tastants. In this case, the increasing response to bitterness (+) in low quantities of PAEE (10–100 mg) appears to be caused by the increase in electric charges of the sensor membranes, and the decreasing response in high quantities of PAEE appears to be caused by the decrease in hydrophobicity of the membranes. On the other hand, the responses to aspartame only showed a monotonic increase in PAEE quantity dependence (100–500 mg). These responses are expected to increase monotonically with increasing PAEE quantity from 500 mg to a certain extent. Hence, the PAEE quantities over 500 mg were chosen as the quantities of lipid in the sensor membranes fabricated in the next stage.

### 3.3. Effect of Lipid Quantity on CPA Values (the Second Stage)

Subsequently, we investigated the effect of a high quantity of PAEE. Simultaneously, we also investigated the aspartame concentration dependence of the sensor responses. The basic taste samples and four additional concentrations of aspartame were measured using the lipid/polymer membranes comprising PAEE (500, 700 or 1,000 mg), PVC and BEO. The responses of these membranes to bitterness (+) and 0.1–10 mM aspartame are shown in Figure 11. The responses of all three membranes to bitterness (+) were less than 5 mV, that is, they were negligible responses. On the other hand, the responses to aspartame (0.1–10 mM) depended on the concentration of aspartame. The responses to 10 mM aspartame were about 20 mV in each membrane. Hence, it is not expected that increasing the PAEE quantities from 1000 mg causes the increase in response to aspartame, rather, it is expected that the aspartame responses will decline with increasing PAEE. Since only the membrane containing 500 mg PAEE showed the clear response of over 20 mV to 10 mM aspartame, we chose the lipid/polymer membrane comprising 500 mg PAEE, PVC and BEO as the sensor membrane for positively charged sweeteners. The responses to aspartame were as follows: CPA value: Y = 8.4 ln X + 1.7, |R| = 0.977, using 1–10 mM range (X: concentration of aspartame (mM); Y: CPA value (mV); relative value: Y = 25 ln X + 13, |R| = 0.993, using 1–10 mM range).

## 4. Conclusions

A sweetness sensor that selectively responds to aspartame was developed in this study. An aim of this study was to expand the measurable region of the taste-sensing system, particularly so that it could measure the sweetness of high-potency sweeteners. As a result, we concluded that the sensor developed in this study had satisfactory performance as a prototype model of the sensor for aspartame. The results show that the sensor achieves the three required specifications (a response of at least 20 mV to 10 mM aspartame in terms of the CPA value, no response to any other basic tastes and the concentration dependence of the responses to aspartame) and has satisfactory selectivity and response for trial use. Future tasks for this sensor are to utilize the relative values as well as CPA values of the sensor for evaluating sweetness and to investigate the behavior of the sensor in real formulation or beverage samples.

## Figures and Tables

**Table 1.** Basic taste samples.

**Taste Sample**	**Components**
Reference solution (RS)	30 mM KCl, 0.3 mM tartaric acid
Saltiness	300 mM KCl, 0.3 mM tartaric acid
Sourness	30 mM KCl, 3 mM tartaric acid
Umami	10 mM sodium glutamate + RS
Bitterness (+)	0.1 mM quinine hydrochloride + RS
Bitterness (−)	0.01 vol% iso-alpha acid + RS
Astringency	0.05% tannic acid + RS
Sweetness	1 M sucrose + RS
Saccharin sodium	5 mM saccharin sodium + RS
Acesulfame potassium	10 mM acesulfame potassium + RS
Aspartame	10 mM aspartame + RS
